# Vision-Based Real-Time Traversable Region Detection for Mobile Robot in the Outdoors

**DOI:** 10.3390/s17092101

**Published:** 2017-09-13

**Authors:** Fucheng Deng, Xiaorui Zhu, Chao He

**Affiliations:** Harbin Institute of Technology (Shenzhen), Shenzhen 518055, Guangdong, China; fuchengdeng@hit.edu.cn (F.D.); hithechao@gmail.com (C.H.)

**Keywords:** binocular sensor, traversable region detection, mobile robot, outdoors

## Abstract

Environment perception is essential for autonomous mobile robots in human-robot coexisting outdoor environments. One of the important tasks for such intelligent robots is to autonomously detect the traversable region in an unstructured 3D real world. The main drawback of most existing methods is that of high computational complexity. Hence, this paper proposes a binocular vision-based, real-time solution for detecting traversable region in the outdoors. In the proposed method, an appearance model based on multivariate Gaussian is quickly constructed from a sample region in the left image adaptively determined by the vanishing point and dominant borders. Then, a fast, self-supervised segmentation scheme is proposed to classify the traversable and non-traversable regions. The proposed method is evaluated on public datasets as well as a real mobile robot. Implementation on the mobile robot has shown its ability in the real-time navigation applications.

## 1. Introduction

In recent decades, the robotics community has made great efforts to develop mobile robots with complete autonomy. Traversable region detection is one of the fundamental problems for such autonomous navigation systems. Numerous vision-based approaches have been proposed for structured road detection. In recent years, some researchers have attempted to tackle more challenging unstructured road conditions where unstructured roads are referred to the roads that have arbitrary surfaces and various shapes without painted markers or distinguishable borders [1]. However, due to the demand of good tradeoff between time efficiency and accuracy, it is still challenging for a ground robot to autonomously locate a variety of traversable areas and safely navigate itself with respect to human-defined rules (i.e., keep off the non-road area) in real-time [2].

Kong et al. [3] proposed a method to decompose the detection process into two steps: vanishing point estimation and road segmentation based upon the detected vanishing point. This approach could be used to detect various types of roads. However, it was limited by high computational complexity of the vanishing point estimation. Many algorithms have attempted to speed up the procedure of vanishing point estimation. Moghadam et al. [4] proposed an optimal local dominant orientation method using joint activities of only four Gabor filters and an adaptive distance-based voting scheme for estimation of the vanishing point. Miksik [5] investigated a method of expanding Gabor wavelets into a linear combination of Haar-like functions to perform fast filtering, and using superpixels in the voting scheme to speed up the process. Besides the vanishing point based road detection methods, many methods have directly differentiated the road pixels from the background using appearance models [1,6,7,8,9]. Tan et al. [6] adopted multiple color histograms to capture variability of the road surface and a single-color histogram to model the background in RGB space. Ramstrom and Christensen [7] proposed to construct Gaussian mixture models (GMMs) for the road and background using UV color, normalized red and green, and luminance components. These methods required the color models to be trained off-line and thus could not be adaptive to the appearance variations in real road conditions. There are also several methods building the model directly from sample regions in the input image. Álvarez and Ĺopez [8] employed normalized histogram of the surrounding area of a set of seed pixels at the bottom part of the image to build the road model using illuminant invariance space. Similarly, Lu [9] selected a region from the bottom part of the input image as road masks to model the road and the background combining complementary color features, such as RGB, Lab and SILTP (Scale Invariant Local Ternary Patterns) texture feature. The performance of these methods depended on the quality of constant sample regions that might be a false road sample area. Instead of selecting a constant sample area, Miksik et al. [10] used a trapezoid at the bottom and center of the image as the initial sample region, and then refined it using the vanishing point. In recent years, different hybrid methods have been proposed to further increase accuracy and robustness [1,11]. In [1], a sample region was first determined using the vanishing point, the geometric and color features of road. Then a model combining color, edge and shape features was used to detect unmarked roads. Álvarez et al. [11] introduced road priors (geographical information) and contextual cues (horizon lines, vanishing points, road markings, 3D scene layout and road geometry) to detect road areas in varying conditions. All of the methods mentioned above, however, are still computationally expensive.

Most recently, Lee et al. have made efforts to reduce the computational load for online applications [12]. In [12], to estimate the traversability in complex and unknown environments for an autonomous vehicle, a self-supervised online learning architecture based on incremental nonparametric Bayesian clustering was developed. This method consists of three stages: superpixel labeling with inertial measurement unit (IMU) and wheel encoder data, incremental cluster learning, and traversable region classification with k-nearest neighborhood. The whole algorithm could be used on a robot platform for traversability estimation in complex and unknown environments as the authors claimed. However, it took about 48.3 s for the incremental clustering leaning and 0.02 s for the traversabilty estimation, which is still far from the real-time requirement of most robotics applications (e.g., at frame rate 30 fps).

It is worth noting that deep learning methods are also popular for traversable region segmentation [13,14,15,16,17]. Xiao et al. [13] presented a road detection algorithm based on structured random forest, making use of the contextual information. The computational time of their algorithm was, on average, 70 ms (Intel Core i5-3230 CPU@2.6GHz) for unstructured road detection. Alvarez et al. [14] used a convolutional neural network based algorithm to learn features for road scene segmentation. Mohan [15] developed a new deep learning architecture, deep deconvolutional neural networks, for road parsing. This method provides one of the best results on the KITTI-Road benchmark. Howeverm the running time was over 2 s (Multiple GPU@2.5GHz). Recently, Oliveira et al. [16] proposed an efficient deep model for monocular road segmentation which obtained the best trade-off between segmentation quality and runtime on the KITTI dataset. However, the runtime was still over 80 ms (NVIDIA Titan X GPU). On the other hand, these deep learning based methods are only capable of being implemented on high-power GPUs which are usually unavailable on a mobile robotic platform. Thus, deep learning based methods are still very challenging for real-time applications at interactive frame rates in a mobile robot.

In this paper, we propose a novel method of detecting traversable region for a mobile robot from a single image in real-time without dependency on any specific hardware. A fast and robust multivariate Gaussian model combining RGB, illumination invariant space (IIS) [18] and local binary pattern (LBP) [19] feature is built from an adaptive sample region determined by the constraints of vanishing point and two road borders. The segmentation could be implemented in real-time on standard CPU equipped by an ordinary mobile robot. The main contributions of this paper are as follows:

- A new method is proposed to robustly estimate a vanishing point, which outperforms the state-of-the-art considering tradeoff of time efficiency and accuracy. The vanishing point is detected by voting of a few line segments formed with some dominant pixels rather than by voting of all pixels in most existing methods.
- A fast, self-supervised segmentation scheme is proposed for unstructured traversable region detection. An appearance model based on multivariate Gaussian is constructed from the sample region adaptively determined by the vanishing point and dominant borders in the input image. This scheme allows real-time performance on a mobile robot.

The remainder of the paper is organized as follows. The proposed method is described in detail in Section 2. Experimental results and discussion are presented in Section 3. Finally, a conclusion is drawn in Section 4.

## 2. Methods

### 2.1. Traversable Region Detection

**Figure 1:**
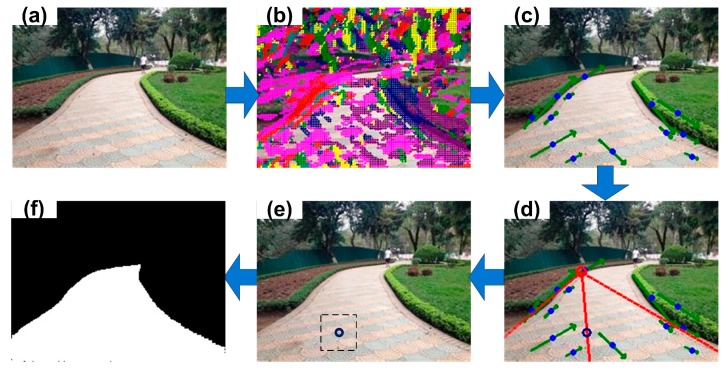
Pipeline of the traversable region detection: (**a**) input image, (**b**) estimating texture orientations (represented by different colors) using Gabor filters, (**c**) grouping dominant pixels with the same orientation into line segment candidates (in green with a blue point), (**d**) estimating the vanishing point (red circle) by the voting of line segment candidates and locating the seed pixel (blue circle), (**e**) selecting a sample region surrounding the seed pixel and constructing an appearance model, (**f**) classifying the pixels into traversable/non-traversable area.

Figure 1 depicts the pipeline of the proposed traversable region detection. First, texture orientation is computed using Gabor filter with eight directions for each pixel of the input image. Secondly, instead of directly using all the pixels to vote for the vanishing point based on their orientations, we only group some dominant pixels with the same orientation into line segment candidates. Thirdly, the vanishing point is estimated by voting of those line segment candidates, and a seed pixel belonging to the road area is located through the constraints of the vanishing point and two road border candidates. Lastly, a sample region surrounding the seed pixel is selected to model the road combining RGB, IIS and LBP features using multivariate Gaussian such that the road can be classified from the background based on this appearance model.

### 2.2. Vanishing Point Estimation

In this paper, Gabor filters are used to estimate local dominant orientation for each pixel because of their well-known accuracy. A 2D Gabor filter for an orientation $\theta_{n}$ and radial frequency *ω* is defined as follows [20],

(1) $$\psi_{\omega ,\theta_{n}}(x,y)=\frac{\omega }{\sqrt{2\pi }c}e^{-\frac{\omega^{2}}{8c^{2}}(4a^{2}+b^{2})}(e^{ia\omega }-e^{-c^{2}/2})$$

where $a=x\cos\theta_{n}+y\sin\theta_{n}$, $b=-x\sin\theta_{n}+y\cos\theta_{n}$, c=π/2, ω=2π/λ and λ is set to $4\sqrt{2}$.

Let I(p) be the grayscale value of input image at p(x,y). The convolution of input image I(p) and a bank of Gabor filters with orientation $\theta_{n}$ and radial frequency *ω* are calculated as,

(2) $$\begin{array}{l} \Gamma_{\theta_{n}}(p)=I(p)⊗\psi_{\omega ,\theta_{n}}\\ \theta_{n}=(n-1)\pi /N,\;n=1,2,\ldots N \end{array}$$

where *N* is the total number of orientations. The square norm of the complex Gabor filter response is then computed as,

(3) Eθn(p)=Re(Γθn(p))2+Im(Γθn(p))2.

Thus, the local dominant texture orientation $\theta_{\max}$ is defined as the orientation corresponding to the strongest Gabor response across all the orientations. More precise angular resolution can be achieved with a larger number of orientations (N=36 in [3]). However, it would be at the cost of computation complexity. In this paper, only 8 orientations are preferred to be used with a resolution of 22.5°. A confidence-rated technique similar to the work of Kong et al. [3] is used to provide a confidence level for the local texture orientation $\theta_{\max}(p)$ at pixel p. Suppose $E_{1}(p)>\ldots >E_{8}(p)$ is the ordered values of Gabor response for the 8 predefined orientations, the confidence in the orientation $\theta_{\max}(p)$ is given by,

(4) $$conf_{\theta_{\max}}(p)=100(1-\frac{1}{5}\sum_{i=2}^{6}E_{i}(p)/E_{1}\left(p\right)).$$

The pixels with a confidence level smaller than a threshold $T_{th}$, i.e., $conf_{\theta_{\max}}<T_{th}$ will be discarded. In our experiments, the optimal $T_{th}$ is set to 35.

It is found out that in many paved roads, those pixels contributing more to the voting share have similar orientations and can be grouped into line segments. Therefore, we propose to first group those dominant pixels into line segment candidates and then use these line segments to vote for the vanishing point.

Instead of using gradient angle like the existing approach [21], the texture orientation is used in this paper under the concept of line-support region for line segment detection. A region growing algorithm is applied to group connected pixels (8-connected neighborhood used in this paper) with a constant orientation tolerance into line-support region. A small orientation tolerance will result in too narrow line-support regions while a large one tends to include too many outliers. Hence, in our experiments, the orientation tolerance is empirically set as 22.5°. Small line-support regions are rejected by the following criterion [21],

(5) $$n_{reg}<-\log_{10}(11{\left(X_{m}Y_{m}\right)}^{5/2})/\log_{10}(\theta_{th}/180)$$

where $n_{reg}$ is the number of pixels in the region, $X_{m}$ and $Y_{m}$ are the sizes of the input image, $\theta_{th}$ is the resolution of each orientation. Once a line-support region $R_{i}$ is found, a least-square-fitting is applied to obtain a line $l_{i}$. A line-support region $R_{i}$ (a set of pixels) must be associated with a line segment, actually a narrowed rectangle with its length and width. Thus, a rectangular approximation should be constructed to evaluate the fitted line. In this paper, we use the center of mass as the center of the rectangle $\left(c_{i,x},c_{i,y}\right)$,

(6) $$\begin{array}{l} c_{i,x}=\sum_{j\in R_{i}}g(j)x(j)/\sum_{j\in R_{i}}g(j),\\ c_{i,y}=\sum_{j\in R_{i}}g(j)y(j)/\sum_{j\in R_{i}}g(j), \end{array}$$

where g(j) is the gradient magnitude of pixel j. The main direction of the rectangle is set to the direction of the fitted line $l_{i}$. Then, the width $L_{i,w}$ and length $L_{i,l}$ of the rectangle are set to the smallest values so as to cover the full line-support region $R_{i}$. The fitted line $l_{i}$ (and the line-support region $R_{i}$) will be rejected if the rectangle is not narrow enough according to the ratio of length to width,

(7) $$r_{i}=L_{i,l}/L_{i,w}\leq r_{th},$$

where $r_{th}$ is a threshold and set to 2 in our experiment.

**Figure 2:**
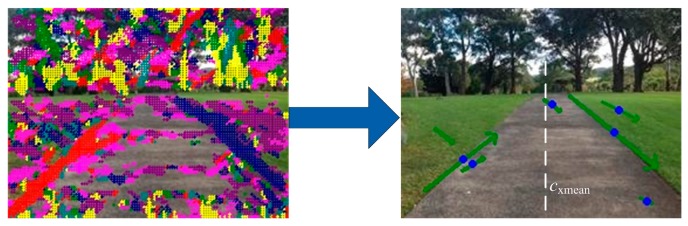
An example of grouping dominant pixels into line segment candidates. Left: image overlaid with texture orientations, 22.5°-pink, 45°-red, 67.5°-cyan, 90°-yellow, 112.5°-green, 135°-deep blue, 157.5°-purple. Right: selected line segment candidates (arrowed green lines with blue points in the middle); the one on the most left is abandoned.

To further evaluate the fitted line $l_{i}$ and select better line segment candidates for the vanishing point estimation, more constraints need to be considered. As Figure 2 shows, after the region growing and line fitting are applied, only a few line segments are extracted. Other less important line segments will be further excluded and could be viewed as noise for the vanishing point estimation. Suppose the set of line segments is ${\left\{l_{i}\right\}}_{M}$ and their slopes and centers are ${\left\{K_{i}\right\}}_{M}$,${\left\{\left(c_{i,x},c_{i,y}\right)\right\}}_{M}$, respectively. It is assumed that the vanishing point is not located on the left/right edges of the image. Only those line segments satisfying the following criterion are selected as candidates for vanishing point estimation:

(8) $$\gamma_{i}=(c_{i,x}-c_{xmean})\cdot K_{i}>0,$$

where $\gamma_{i}$ is a flag for line $l_{i}$, $c_{xmean}$ is the average of all the line centers in the x direction, $c_{xmean}=\sum_{i=1}^{M}c_{i,x}/M$, and M is the total number of lines.

Finally, a set of line segment candidates ${\left\{l_{i}\right\}}_{M}$ obtained above will be used to estimate the vanishing point by a weighted voting scheme. A distance parameter $D_{i,j}$ is defined for voting,

(9) $$D_{i,j}=\sum_{k}\exp(-\frac{L_{i,l}+L_{j,l}}{L_{l}})d_{i,j,k},$$

where $L_{i,l}$ and $L_{j,l}$ are the length of lines $l_{i}$ and $l_{j}$ respectively, $L_{l}$ is the sum of the length of all the lines, $L_{l}=\sum_{i=1}^{M}L_{i,l}$, $d_{i,j,k}$ is the distance from the intersection point of the lines $l_{i}$ and $l_{j}$ to the line $l_{k}$ in the ${\left\{l_{i}\right\}}_{M}$.

Thus, the intersection point $p_{vp}(x_{i,j},y_{i,j})$ corresponding to $\underset{p(x_{i,j},y_{i,j})}{\arg\min}D_{i,j}$ is selected as the vanishing point. The complete algorithm for vanishing point estimation can be summarized as Algorithm 1.

**Algorithm 1.** Vanishing Point Estimation1.Compute the local dominant texture orientation $\left\{\theta_{\max}(p)\right\}$ at each pixel p using Gabor filter with 8 orientations2.Group dominant pixels into line segments ${\left\{l_{i}\right\}}_{M}$2.1.Find a line-support region $R_{i}$ using region growing with 8-connected neighborhood based on the texture orientation of each pixel.2.2.*if* $n_{reg}<-\log_{10}(11{\left(X_{m}Y_{m}\right)}^{5/2})/\log_{10}(\theta_{th}/180)$ reject the line-support region $R_{i}$*else* get a line $l_{i}$ using least-square-fitting2.3.Construct a rectangular approximation for $R_{i}$2.4.*if* $r_{i}=L_{i,l}/L_{i,w}\leq r_{th}$ reject the line $l_{i}$*else* add the line $l_{i}$ into ${\left\{l_{i}\right\}}_{M}$3.Refine the line segment candidates ${\left\{l_{i}\right\}}_{M}$for each line $l_{i}$*if* $\gamma_{i}=(c_{i,x}-c_{xmean})\cdot K_{i}>0$ keep it*else* abandon it
4.Vote for the vanishing point $p_{vp}(x_{i,j},y_{i,j})$ using ${\left\{l_{i}\right\}}_{M}$4.1.Compute the distance parameter $D_{i,j}=\sum_{k}\exp(-\frac{L_{i,l}+L_{j,l}}{L_{l}})d_{i,j,k}$ for the intersection point $p(x_{i,j},y_{i,j})$ of arbitrary lines $l_{i}$ and $l_{j}$ in ${\left\{l_{i}\right\}}_{M}$4.2.Find the intersection point $p_{vp}(x_{i,j},y_{i,j})=\underset{p(x_{i,j},y_{i,j})}{\arg\min}D_{i,j}$ i.e., the estimated vanishing point.

### 2.3. Sample Region Selection

Since the appearance of traversable region varies significantly, it is more plausible to build the appearance model adaptively with the input image than with off-line training images [1]. To this end, the selection of a sample region of the road plays an important role. The selected sample region tends to include non-traversable region with non-adaptive methods. In our approach, the detected vanishing point is used to adaptively define the sample region because it provides a strong clue to the true location of road area.

**Figure 3:**
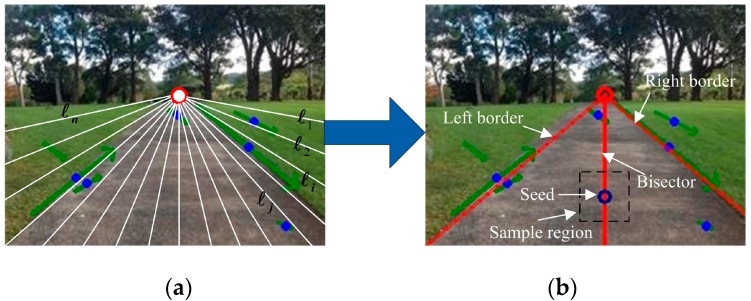
Sample region selection for constructing an appearance model. (**a**) image with 17 evenly distributed imaginary rays (in white) originating from the vanishing point. (**b**) image with dominant borders (in red) and sample region surrounding the seed (blue circle).

In this paper, a similar technique is used as presented in Ref. [3] to find the two most dominant borders from a set of imaginary rays that originate from the initially estimated vanishing point. The difference is that we just roughly estimate the borders to define the sample region rather than to segment the traversable area. Specifically, we only consider 17 evenly distributed imaginary rays with the angle between two neighboring ones being 10°, as is shown in Figure 3. Suppose $A_{i,L}$ and $A_{i,R}$ are two neighboring regions on either side of the ray $\ell_{i}$ respectively. The color difference of $A_{i,L}$ and $A_{i,R}$ for each channel of color space (RGB used in this paper) is defined as,

(10) $$\Delta {\left(A_{i,L},A_{i,R}\right)}_{c}=\frac{\left|\mathrm{mean}(A_{i,L})-\mathrm{mean}(A_{i,R})\right|}{\sqrt{\mathrm{var}(A_{i,L})+\mathrm{var}(A_{i,R})}},$$

where mean() and var() are the mean and variance of pixel value in the region. Let $\Delta (A_{i,L},A_{i,R})=\max\left\{\Delta {\left(A_{i,L},A_{i,R}\right)}_{c}|{}_{c=R,G,B}\right\}$, then the right and left borders are simply defined as the ray $\ell_{j}$ and $\ell_{k}$ to satisfy the following expressions,

(11) $$\begin{array}{l} \Delta (A_{j,L},A_{j,R})=\max(\Delta (A_{i,L},A_{i,R})|{}_{i=2,3,\ldots 8}),\\ \Delta (A_{k,L},A_{k,R})=\max(\Delta (A_{i,L},A_{i,R})|{}_{i=9,10,\ldots 16}). \end{array}$$

Once the right and left borders are obtained, a new imaginary ray $\ell_{b}$ is constructed to be the bisector of ray $\ell_{j}$ and $\ell_{k}$. A seed point $p_{\mathrm{seed}}$ is found at the location of 2/3 of the bisector. Finally, a region $R_{S}$ of K×K (K=15 in our experiments) surrounding the seed is selected as the sample region, as shown in the right image of Figure 3.

### 2.4. Segmentation Method

In this paper, a computationally efficient model based on multivariate Gaussian with complementary features, such as RGB, IIS and LBP, is used for segmentation. Given the sample region, the mean feature vector $\mu_{C}$ and the covariance matrix $\sum {}_{C}$ are first obtained using 7 channels of each pixel,

(12) $$\begin{array}{l} \mu_{C}=\frac{1}{n_{s}}\sum_{i=1}^{i=n_{s}}p_{C,i},\\ \sum {}_{C}=\frac{1}{n_{s}}\sum_{i=1}^{i=n_{s}}p_{C,i}p_{C,i}^{T}-\mu_{C}\mu_{C}^{T},\\ C=\{r,g,b,c_{1},c_{2},c_{3},c_{LBP}\}. \end{array}$$

where $n_{s}$ is the total number of pixels in the sample region, $p_{C,i}$ is the value of ith pixel for channel C (r,g,b are for RGB space, $c_{1},c_{2},c_{3}$ are for IIS space, and $c_{LBP}$ is for LBP).

Then, the likelihood of a pixel p belonging to the road/non-road region is measured as the Mahalanobis distance between the pixel and the learned model,

(13) $$D(p)=\sqrt{{\left(p-\mu_{C}\right)}^{T}\sum {}_{C}^{-1}(p-\mu_{C})}.$$

For all the pixels in the sample region, the initial mean $\mu_{D_{0}}$ and the variance $\sigma_{D_{0}}$ are computed as,

(14) $$\begin{array}{l} \mu_{D_{0}}=\frac{1}{n_{s}}\sum_{i=1}^{i=n_{s}}D(p_{i}),\\ \sigma_{D_{0}}=\sqrt{\frac{1}{n_{s}}\sum_{i=1}^{i=n_{s}}{\left(D(p_{i})-\mu_{D_{0}}\right)}^{2}}. \end{array}$$

A pixel $p_{j}$ is classified into the road region if it satisfies the following condition,

(15) $$\left|D(p_{j})-\mu_{D_{k}}\right|<\lambda \sigma_{D_{k}}$$

where λ is a parameter depending on the location of the pixel. $\mu_{D_{k}}$ and $\sigma_{D_{k}}$ are respectively the mean and variance of Mahalanobis distance for all the pixels in the road region. $\mu_{D_{k}}$ and $\sigma_{D_{k}}$ will be adaptively updated as the new road pixel outside the sample region is found.

**Figure 4:**
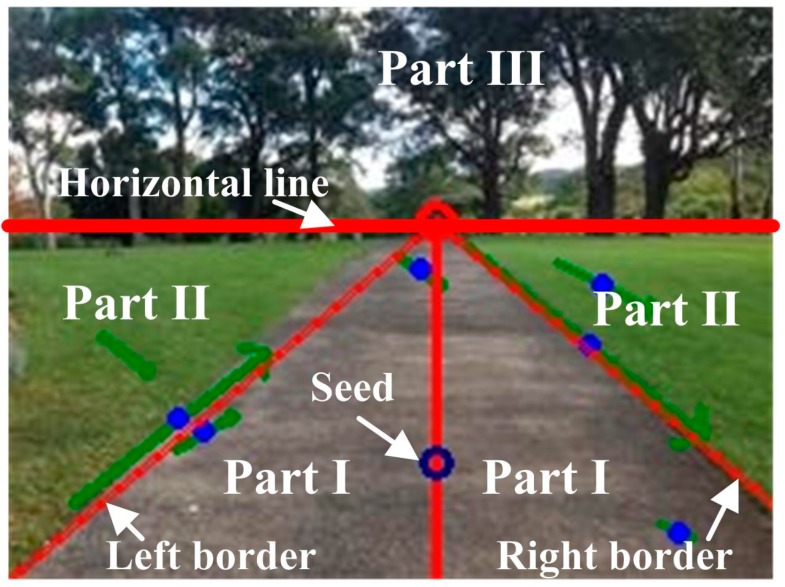
Traversable region segmentation starting from the seed with region growing. The region between left and right border is Part I; the region above horizontal line is Part III and the rest of the image part is Part II.

In this paper, the segmentation process starts from the seed pixel with region growing method (8-connected neighborhood), as shown in Figure 4. The whole image is divided into three parts by the border candidates and the horizontal line on which the vanishing point is located. To make the segmentation be accurate and robust to the noise, we apply adaptive threshold by changing the parameters λ, $\mu_{D_{k}}$ and $\sigma_{D_{k}}$. The parameter λ changes with the pixel location because the likelihood of a pixel belonging to the road region varies for different areas of the image. In our experiments, λ is set as,

(16) $$\lambda =\left\{ \begin{array}{l} 3,\;if\;p_{j}\in \mathrm{Part}\;I,\\ 1,\;if\;p_{j}\in \mathrm{Part}\;\mathrm{II},\\ 0.5,\;if\;p_{j}\in \mathrm{Part}\;\mathrm{III}. \end{array}\right.$$

Furthermore, once a new road pixel $p_{j}$ is found and added to the road region, the parameters $\mu_{D_{k}}$ and $\sigma_{D_{k}}$ are updated as,

(17) $$\begin{array}{l} \mu_{D_{k}}=[\mu_{D_{k-1}}n_{s,k-1}+D(p_{j})]/(n_{s,k-1}+1),\\ \sigma_{D_{k}}=\sqrt{\left[\sigma_{D_{k-1}}^{2}n_{s,k-1}+{\left(D(p_{j})-\mu_{D_{k}}\right)}^{2}]/(n_{s,k-1}+1\right)}, \end{array}$$

where $n_{s,k-1}$ is the total number of pixels in the current road region, specifically, $n_{s,0}$ is the total number of pixels in the sample region.

The segmentation algorithm is summarized as Algorithm 2.

**Algorithm 2.** Traversable Region Segmentation1.Find the seed pixel $p_{\mathrm{seed}}$ and the sample region $R_{S}$ surrounding the seed by the vanishing point.2.For each pixel $p_{i}\in R_{S}$ on each channel, compute the mean feature vector $\mu_{C}$ and covariance matrix $\sum {}_{C}$.3.For each pixel $p_{i}\in R_{S}$, compute Mahalanobis distance $D(p_{i})$ and then compute the initial mean $\mu_{D_{0}}$ and variance $\sigma_{D_{0}}$4.Start segmentation from the seed pixel $p_{\mathrm{seed}}$ with region growing4.1.add $p_{\mathrm{seed}}$ to the traversable region $R_{\mathrm{trav}}$,4.2.for each $p_{i}\in R_{\mathrm{trav}}$ do for each $p_{j}$ neighbor of $p_{i}$ and $status(p_{j})\neq \mathrm{used}$ do if $\left|D(p_{j})-\mu_{D_{k}}\right|<\lambda \sigma_{D_{k}}$  add the pixel $p_{j}$ to $R_{\mathrm{trav}}$,  update parameters $\mu_{D_{k}}$ and $\sigma_{D_{k}}$,  $\mathrm{status}(p_{j})=\mathrm{used}$  end endend

## 3. Experimental Results and Discussion

Three experiments have been conducted to evaluate the proposed method. Firstly, vanishing point detection was tested on an image dataset for unstructured pedestrian lane detection and vanishing point estimation (PLVP) [1]. This dataset consists of 2000 images of unstructured lanes under various environmental conditions. Another more challenging image dataset from Ref. [3] (referred to as Challenge dataset in the following section) was also used for more intensive tests. This Challenge dataset contains 1003 images in total including 430 images taken along a Grand Challenge route in Southern California desert. All the images were normalized to the same size 240 × 180 and all the algorithms were run on a standard personal laptop (Intel i5-3230 CPU) without optimization or GPU acceleration. Then, the traversable region segmentation was evaluated on the PLVP dataset as well as KITTI road benchmark [22] to demonstrate the performance of the proposed method on different unstructured scenarios. Lastly, to show the effectiveness of the method for real-time application on robotics, the whole proposed framework was implemented on a Summit XL mobile robot platform with a binocular sensor (baseline 7 cm) in an unstructured campus environment.

### 3.1. Vanishing Point Detection

To evaluate the performance of vanishing point estimation algorithm, we compare the proposed method with two other related methods. One comparable method is a Gabor-based method presented by Kong et al. [3]. In this method, the vanishing point was estimated directly by the voting of all pixels based on their local orientations that were computed using Gabor filters in 36 directions. MATLAB source codes provided by the authors of Ref. [3] were implemented for comparison. The other comparable method is a Hough-based method proposed by Wang et al. [23]. In this method, Hough transform was first used to extract line segments on the edge map. Then, the vanishing point was detected by voting the intersections of line pairs. This method was implemented with C++ by us since the source code is not publicly available.

To quantitatively assess the vanishing point estimation, the estimation error is defined as follows [4],

(18) $$\delta_{\mathrm{vp}}=\left|p_{d}-p_{g}\right|/L,$$

where $p_{d}$ and $p_{g}$ are the detected vanishing point and ground-truth respectively, L is the diagonal length of the image.

**Figure 5:**
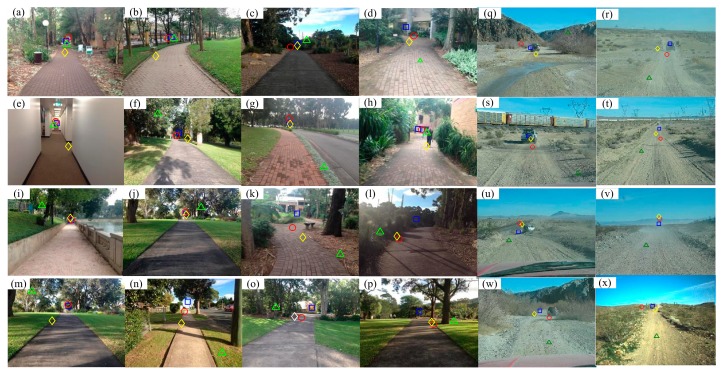
Examples of vanishing point estimation with different methods. Ground truth: blue square, proposed method: red circle, Gabor-based method [3]: yellow rhombus, Hough-based method [23]: green triangle ((**a**–**p**) from PLVP dataset, (**q**–**x**) from Challenge dataset).

Some examples of vanishing point estimation with different methods are shown in Figure 5. Table 1 shows the vanishing point estimation performances both on the PLVP dataset and Challenge dataset with different methods in terms of accuracy and runtime.

According to Figure 5, the Gabor-based method was easily affected by clutter pixels in an image with a complex background (e.g., Figure 5b,e,k,m) because all pixels in a half-disk region were directly used to vote for the vanishing point candidates. In contrast, the proposed method just takes those dominant pixels that contribute more for the voting of vanishing point candidates and thus is more robust to clutter noisy pixels. Thus, the proposed method has a better performance than the Gabor-based method on the PLVP dataset. However, only a few line segment candidates are utilized to estimate the vanishing point with a simple voting scheme in the proposed method. Too few candidates will affect the estimation accuracy especially in very challenging scenarios (e.g., desert regions Figure 5q–x). Contrarily, the Gabor-based method outperforms the proposed one on the Challenge dataset because plenty of voting points are always available for the Gabor-based method. According to Table 1, the average errors of the proposed method on the PLVP dataset and Challenge dataset are 0.0734 ± 0.0858 and 0.1023 ± 0.1085, respectively, while the average errors of the Gabor-based method are 0.0812 ± 0.1042 and 0.0909 ± 0.1010, respectively. It is concluded that these two methods have close performances in terms of accuracy. In addition, the Hough-based method easily failed for natural scenes containing noisy edges or many short line segments (e.g., Figure 5f,g,i,k,m,n,p) because the vanishing point was simply estimated based on all the straight lines extracted from the image. Instead, the proposed method forms pixels into a line segment based on their texture orientations and employs a strict rejection scheme to keep only a small number of valid line segment candidates.

As shown in Table 1, the average computation time of the proposed method was significantly shorter than that of the Gabor-based method. The average error of Hough-based method was much higher than that of the proposed method although it was about two times faster than the proposed method.

In summary, the proposed method can achieve good tradeoff between accuracy and time efficiency for real time implementation.

### 3.2. Traversable Region Segmentation

Two comparable methods were used to make comparisons for road segmentation. One is a boundary-based method presented in Ref. [3] while the other is a pixel-based method presented in Ref. [10].

**Figure 6:**
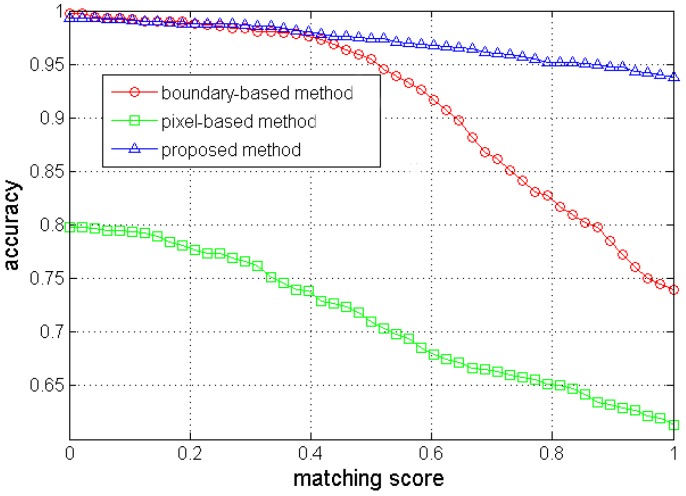
Segmentation accuracy with different methods on the pedestrian lane detection and vanishing point estimation (PLVP) dataset.

To quantitatively evaluate the road segmentation accuracy, we employ a similar approach as the one in Ref. [3]. Suppose that $A_{d}$ is the segmented road area and $A_{g}$ is the binarized ground-truth. The matching score is calculated as,

(19) $$\eta (A_{d},A_{g})=\frac{\left|A_{d}\cap A_{g}\right|}{\left|A_{d}\cup A_{g}\right|}$$

where traversable areas for $A_{d}$ and $A_{g}$ are set to 1 while non-road areas are set to 0. The matching score η can reach maximum value of 1 only when the detected road area completely coincides with the ground-truth. In order to show the road segmentation performance on the dataset, we change the matching score from 0 to 1 and compute the rate of correctly segmented images (Figure 6). Six examples of road segmentation with different methods are demonstrated in Figure 8.

**Figure 7:**
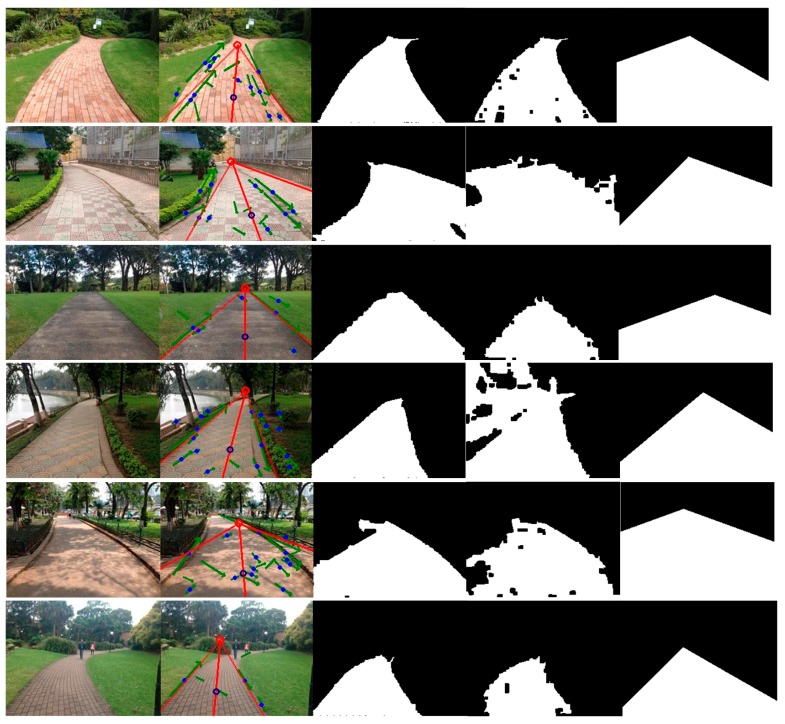
Examples of segmentation. Column 1: input image, Column 2: vanishing point and dominant borders detected by the proposed method, Column 3: segmentation by the proposed method, Column 4: the pixel-based method [10], Column 5: the boundary-based method [3].

According to Figure 6, the proposed method outperforms the other two methods. In the pixel-based method [10], a training region at the bottom of the image was selected to construct GMMs for the road appearance. However, the training region might contain non-road pixels and cannot always represent the true road area. For instance, the segmentation for the image on the sixth row in Figure 7 is not satisfying because the sample region included a small portion of non-road pixels. Moreover, it was difficult to determine an appropriate number of Gaussian models and the threshold for segmentation (e.g., it is over segmented for the image on the fourth row in Figure 7). In contrast, the proposed method tends to select an optimal sample region based on the vanishing point and dominant borders. In addition, a multivariate Gaussian model combining complementary features and an adaptive threshold are used in the proposed method to robustly segment the road from the background.

**Figure 8:**
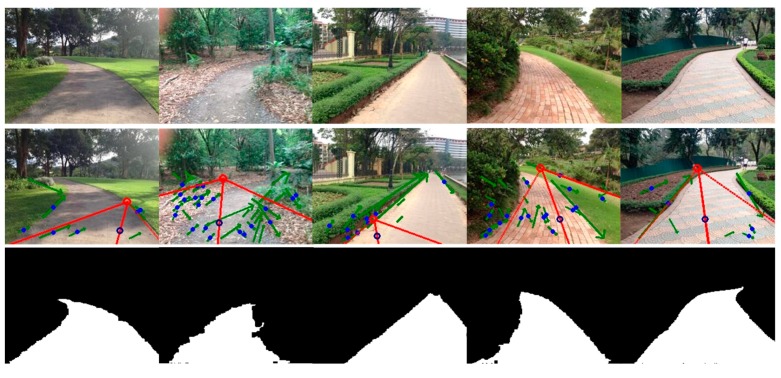
Examples of segmentation with the proposed method under conditions of inaccurate vanishing point and dominant borders. Row 1: input images, Row 2: vanishing point and dominant borders, Row 3: segmentation.

As for the boundary-based method [3], the segmentation depended on detection of the dominant borders. However, inaccurate dominant borders would lead to unexpected road segmentation. For instance, the road segmentation for the image on the first row in Figure 7 includes much of the non-road regions because of false borders. Furthermore, this method classified all the pixels locating in the area between two straight-line borders into road pixels and thus was not suitable for most curved roads (e.g., the segmentations for images of curved roads on the second, fourth, fifth and sixth rows in Figure 7 either included some non-road pixels or excluded some road pixels). In comparison, the proposed method also utilizes the dominant borders but does not strongly depend on them. If there is a big tree or building on the left and near the camera, the edges of the tree-trunk or building might form a line segment and become a line segment candidate used for voting. On the one hand, these kinds of noisy candidates can be rejected at some degrees by the strict criterions (Equations (5), (7) and (8)). Moreover, as can be seen in Figure 8, even though the vanishing point estimation or the dominant border detection was not good enough, a seed pixel of road could still be correctly found. Then, the correct road sample region surrounding the seed could be used to further build the appearance model. Thus, the subsequent segmentation would not be influenced by such inaccurate vanishing points or borders.

**Figure 9:**
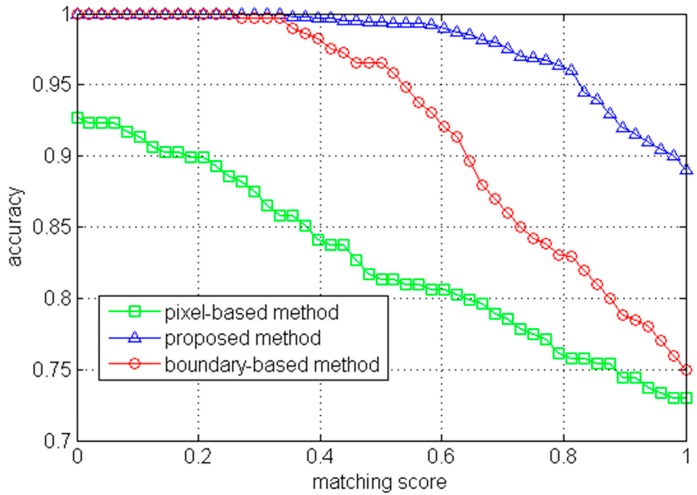
Segmentation accuracy with different methods on the KITTI road benchmark (unmarked lane subset of KITTI).

The KITTI road benchmark consists of urban unmarked, marked, and multiple marked lanes. Because this paper mainly focuses on unmarked traversable region detection, the unmarked lane subset of KITTI was used to further evaluate the performance. According to Figure 9, the proposed method has achieved good performance on this public dataset compared with the other two methods.

The average processing time of the proposed method for traversable region detection (including vanishing point estimation and segmentation) was 28.16 ms, which is significantly faster than that of the pixel-based method [10] (2.93 s) and the boundary-based method [3] (63.56 s). In other words, our algorithm can be run in real-time over 30 fps on standard CPU although the efficiency of the proposed algorithm could be further improved by parallel computing.

### 3.3. Real Time Implementations for Robot Navigation

**Figure 10:**
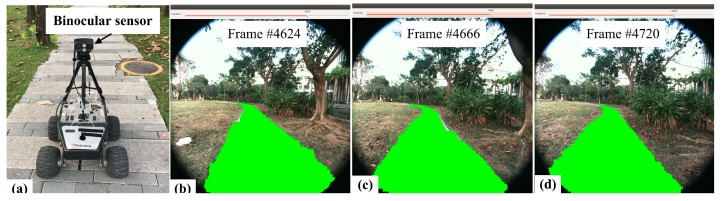
Real-time traversable region detection, (**a**) mobile robot platform with binocular sensor; (**b**–**d**) frame #4624, #4666, #4720 (image frame rate 30 fps).

The proposed framework has been implemented on a real mobile robot in campus environments with unstructured pedestrian lanes (Figure 10). The robot was only allowed to travel on the normal pedestrian lanes like a human being. In our experiments, the lanes are 1.5–2 m wide. The robot must accurately localize itself and simultaneously build the traversable map for the environment.

The depth of the traversable region could be recovered with the binocular images by stereo matching. We adopt a fast stereo matching algorithm suitable for embedded real-time systems described in [24]. Once the disparity for a pixel p(x,y) of traversable region is found by stereo matching, the 3D point in the sensor coordinates can be calculated with,

(20) $$\left(\begin{array}{l} x_{c}\\ y_{c}\\ z_{c} \end{array}\right)=K^{-1}\left(\begin{array}{c} x\cdot z_{c}\\ y\cdot z_{c}\\ z_{c} \end{array}\right),$$

where $z_{c}=b\cdot f/d$, b is the baseline, f is the focal length, d is the disparity and K is the calibration matrix. To get the 3D points $p(x_{w},y_{w},z_{w})$ of traversable region in world coordinates, a transformation needs to be performed according to the current pose of sensor,

(21) $$\left(\begin{array}{c} x_{w}\\ y_{w}\\ z_{w}\\ 1 \end{array}\right)=T^{-1}\left(\begin{array}{c} x_{c}\\ y_{c}\\ z_{c} \end{array}\right),$$

where T=[R|t], R and t are rotation and translation matrix respectively for the consecutive robot pose. In this experiment, ORB-SLAM [25] is chosen to estimate the pose and build the map.

**Figure 11:**
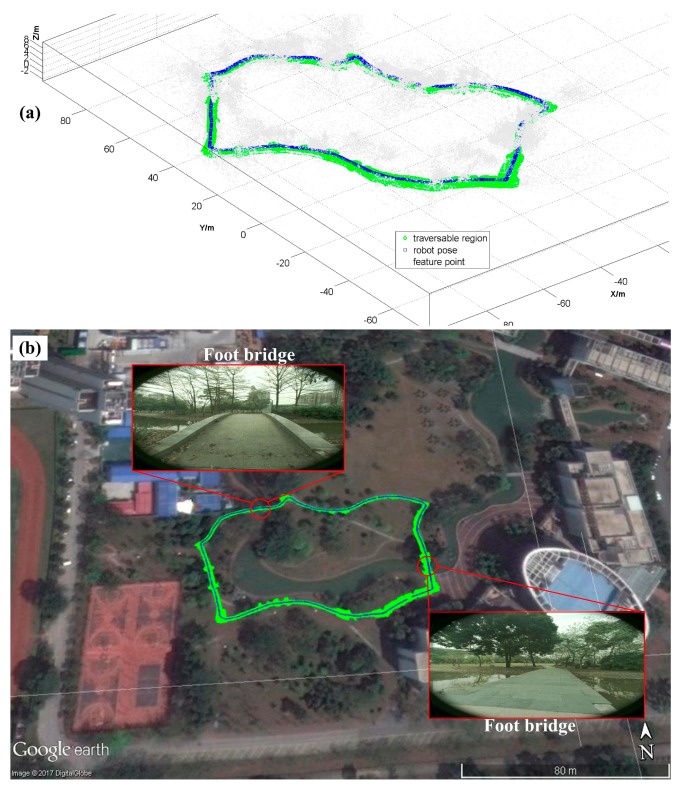
A complete loop trajectory of the robot with traversable regions (**a**) blue: robot pose, green: traversable region, grey: feature point; (**b**) traversable mapping overlapped on real map.

To avoid producing too many 3D points of traversable region and save memory, a voxel filter [26] is used to downsample the point cloud. Figure 11a gives the result of a whole loop trajectory (~240 m) in the experiment. The grey point cloud represents feature points in the map while the green points represent the resulting traversable region. The robot poses and the built map are overlapped on the Google map in Figure 11b. These experimental results have shown that the robot can build a traversable map of the unstructured environment in real-time for robot navigation. For instance, the two narrow foot bridges (~1.5 m wide) on the real trajectory have been accurately mapped so that the robot could correctly locate the bridges and cross them like a human being without falling into the river. More analysis of localization and mapping results will be presented in the future work together with other related algorithms, which is not in the scope of this paper.

## 4. Conclusions

This paper proposes a novel method for real-time detection of unstructured traversable regions from a single image in complex outdoor environments. This method utilizes vanishing point estimated by a new fast voting scheme to adaptively determine a road sample region of input image. A self-supervised segmentation approach based on a multivariate Gaussian model built from the sample region is used to classify the road and background rapidly and robustly. Experimental results on the public dataset have shown that the proposed method is able to detect various unstructured roads in real-time. Furthermore, implementation on a real mobile robot in challenging environments has shown the effectiveness of the proposed framework for robot navigation where the traversable region detection could be performed at a frame rate of 30 fps. Future work will focus on combing the proposed traversable region detection method with new localization and mapping algorithms to facilitate robot navigations in more challenging large-scale environments.

## Figures and Tables

**Table 1:** Vanishing point estimation performance.

Method	Average Error		Times (s)
	PLVP Dataset	Challenge Dataset	
Gabor-based method	0.0812 ± 0.1042	0.0909 ± 0.1010	11.712 ^a^
Hough-based method	0.1463 ± 0.1353	0.2464 ± 0.1464	0.009 ^b^
Proposed method	0.0734 ± 0.0858	0.1023 ± 0.1085	0.021 ^c^

^a^ the code is in MATLAB, ^b,c^ the codes are in C++.

## Supplementary Materials

The following are available online at http://www.mdpi.com/1424-8220/17/9/2101/s1, Video S1: traversable region detection.
